# Quantitative Aortography Analysis of JenaValve’s Trilogy Transcatheter Aortic Valve Implantation System in Patients With Aortic Regurgitation or Stenosis

**DOI:** 10.1016/j.shj.2024.100346

**Published:** 2024-07-23

**Authors:** Tsung-Ying Tsai, Hesham Elzomor, Hendrik Wienemann, Pruthvi Chenniganahosahalli Revaiah, Ralph Stephan von Bardeleben, Alexander Tamm, Scot Garg, Osama Soliman, Yoshinobu Onuma, Hans R. Figulla, Matti Adam, Tanja Rudolph, Patrick W. Serruys

**Affiliations:** a CORRIB Research Centre for Advanced Imaging and Core Laboratory, University of Galway, Galway, Ireland; b Cardiovascular Center, Taichung Veterans General Hospital, Taichung, Taiwan; c Department of Internal Medicine III – Cardiology, University of Cologne, Faculty of Medicine and University Hospital Cologne, Cologne, Germany; d Heart Valve Center, Heart and Vascular Center, Universitätsmedizin Mainz, Mainz, Germany; e Department of Cardiology, Royal Blackburn Hospital, Blackburn, UK; f University Heart Center Jena, Friedrich-Schiller University, Jena, Germany; g Clinic for General and Interventional Cardiology/Angiology, Herz- und Diabeteszentrum NRW, Ruhr-Universität Bochum, Bad Oeynhausen, Germany

**Keywords:** Jenavalve, Paravalvular leak, Pure aortic regurgitation, Transcatheter aortic valve implantation, Video-densitometry

## Abstract

**Background:**

JenaValve’s Trilogy transcatheter heart valve (THV) (JenaValve Inc, Irvine, CA) is the only *conformité européenne*-marked THV system for the treatment of aortic regurgitation (AR) or aortic stenosis (AS). However, its efficacy has not been quantitatively investigated pre- and post-implantation using video-densitometric analysis.

**Methods:**

Using the CAAS-A-Valve 2.1.2 software (Pie Medical Imaging, Maastricht, the Netherlands), an independent core lab retrospectively analyzed the aortograms of 88 consecutive patients (68 severe AR; 20 severe AS) receiving the JenaValve THV in three European centers. Video-densitometric AR was categorized by the regurgitant fraction (RF) as none/trace AR (RF ≤ 6%), mild (6% < RF ≤ 17%), and moderate/severe AR (RF > 17%).

**Results:**

Pre- and post-THV aortograms were analyzable in 17 (22.4%) and 47 (54.0%) patients, respectively. The main reasons preventing analysis were the descending aorta overlap of the outflow tract (30%) and insufficient frame count (6%). The median RF pre- and post-THV implant was 31.0% (interquartile range 21.5-38.6%) and 5.0% (interquartile range 1.0-7.0%, *p* < 0.001), respectively. The post-THV incidence of none/trace AR was 72.3%, and of mild AR, 27.7%, with no cases of moderate/severe AR. Video-densitometry analysis of the 12 AR cases with paired pre- and post-THV showed a reduction in the RF of 21.8% ± 8.1%.

**Conclusions:**

Quantitative aortography confirms the low rates of AR and the large reduction in RF following the implantation of Jenavalve’s Trilogy THV, irrespective of implant indication. However, these limited data need corroborating in prospective studies using standardized acquisition protocols.

## Highlights

• The JenaValve’s Trilogy transcatheter heart valve (THV) system is the only CE-marked THV system for severe aortic regurgitation (AR), but its efficacy has not been evaluated with quantitative aortography analysis.
• Pre- and post-THV aortograms were analyzable in 17 (22.4%) and 47 (54.0%) patients, respectively, with the main reasons preventing analysis being descending aorta overlap of the outflow tract (30%) and insufficient frame count (6%).
• The median post-THV regurgitant fraction was 5.0% (interquartile range 1.0-7.0%), with the incidence of postimplant AR exclusively none/trace or mild and more patients with none/trace than mild.
• There was no significant difference in the severity of AR postimplant between patients treated for severe AR or severe aortic stenosis.

## Graphical abstract

**Figure:**
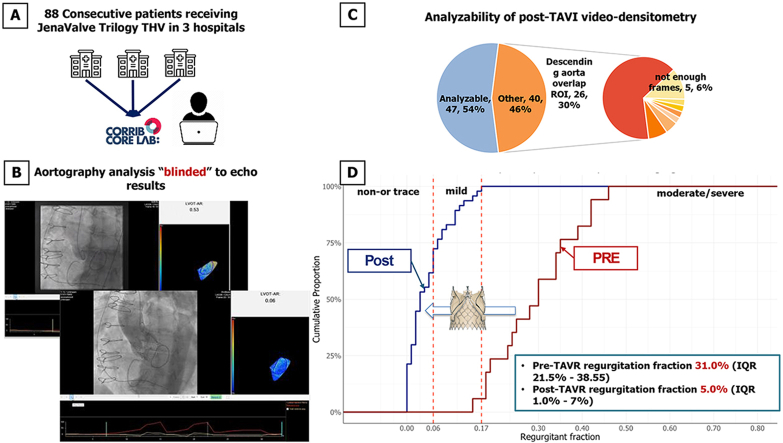

## Background

Severe aortic regurgitation (AR) affects 2% of the elderly population and is associated with significant morbidity and mortality.^1^^,^^2^ AR is a continuous disease process leading to progressive cardiac remodeling from left ventricle (LV) hypertrophy to LV dilatation, followed by systolic and diastolic dysfunction.^3^ If left untreated, mortality rates in patients with severe AR are greater than 10% per-year;^1^ however, early treatment, especially in those with preserved left ventricular ejection fraction, leads to improved long-term survival compared to age-adjusted controls.^1^ Unfortunately, less than a quarter of patients with severe AR receive surgical aortic valve replacement (SAVR) despite its Class IB recommendation in the current American College of Cardiology/American Heart Association guidelines for Valvular Heart Disease,^4^ with the invasiveness of SAVR a commonly cited reason for not performing SAVR. Consequently, a certain proportion of these patients have received off-label treatment with transcatheter aortic valve implantation (TAVI) using devices designed for severe aortic stenosis (AS)^5^^,^^6^; however, large registry datasets suggest that efficacy remains inferior to SAVR, even with newer generation transcatheter heart valves (THVs).^7^

The dedicated JenaValve Trilogy THV (JenaValve Inc, Irvine, CA) is the only CE-marked TAVI system with an indication for both severe AR and AS. Promising short-term outcomes have been reported using this transfemoral THV in patients with severe AR;^8^ however, its efficacy in reducing AR has not been investigated with quantitative analysis.

Quantitative aortography with video-densitometry is an objective, highly reproducible, and accurate tool for quantifying the degree of AR following TAVI,^9^ which has been extensively vetted and validated *in vitro*, *in vivo*, and in clinical settings compared to trans-thoracic echo, trans-esophageal echocardiography, and magnetic resonance imaging. It has been used to evaluate many TAVI devices and is currently being used in major trials and registries.^9^

This retrospective multicenter study aims to quantify the severity of AR using video-densitometry angiography in consecutive patients treated with the JenaValve THV.

## Methods

### Study Design

The study population comprised consecutive patients with severe AS or AR receiving the JenaValve THV in three leading European centers between September 2021 and May 2023. The decision to perform TAVI and the device selection were made by the local heart team and followed standard local protocols at each participating center.^10^ The study was conducted in accordance with the principles of the Declaration of Helsinki and good clinical practice in each participating center.

Pre- and post-THV angiograms were collected, anonymized, and transferred to an independent academic core lab (CORRIB core lab, Galway, Ireland), wherein quantitative video-densitometry was performed by experienced analysts who were blinded to echocardiography results using the CAAS-A-Valve 2.1.2 software (Pie Medical Imaging, Maastricht, the Netherlands). Video-densitometric AR was categorized according to the regurgitant fraction (RF) using previously validated cutoffs as none/trace AR (RF ≤ 6%), mild (6% < RF ≤ 17%), and moderate/severe AR (RF > 17%).^9^ Baseline patient characteristics, procedure details, and in-hospital outcomes were collected prospectively.

### Device Description

The JenaValve’s Trilogy THV system is a self-expanding bioprosthetic valve with a specially designed nitinol frame supporting three porcine pericardial valve leaflets.^11^ The valve’s frame has three locators that help position it in the aortic sinuses and at the base of the native leaflets. After positioning, the nitinol stent arms clip the prosthesis onto the native aortic leaflets. This active clip fixation minimizes the radial forces exerted on the underlying aortic and cardiac structures compared to traditional THV devices, which need to anchor onto calcium in the aortic annulus and thus allow treatment of aortic valve disease without calcification, such as AR. The locators also facilitate paravalvular seals and act in synergy with the 24 conforming sealing rings at the inflow part of the valve. The nitinol stent also has large open cells that enable easy coronary access once implanted. The valve is indicated for use in patients with native symptomatic severe AR or severe AS; notably, for the latter, predilation is required before implantation.

### Statistical Analysis

Categorical variables are presented as counts and percentages, while continuous variables are presented as mean ± SD for normally distributed variables and as median (first and third quartile) for non-normally distributed variables. Categorical variables were compared with the chi-square test or the Fisher exact test, while continuous variables were compared with the t-Student test or the Mann-Whitney U test as appropriate. The Shapiro-Wilk test was used to test for normal distribution. All statistical analysis was performed using R version 4.2.1 (R Foundation for Statistical Computing, Vienna, Austria). A 2-sided *p* value <0.05 was considered statistically significant.

## Results

### Patient and Procedure Characteristics

The baseline characteristics of the 88 consecutive patients receiving a JenaValve THV between September 2021 and May 2023 are shown in Table 1. Their mean age was 77.8 ± 8.6 years, with 39 (44.3%) females. The indication for treatment was severe AR in 68 (77.3%) patients and severe AS in 20 (22.7%). The median EuroSCORE II and Society of Thoracic Surgeons risk scores were 3.79 [2.11, 7.13] and 2.63 [1.71, 4.24], respectively. Most patients were symptomatic with New York heart association class III/IV dyspnea (n = 63, 71.6%). The demographics, surgical risks, and comorbidities were similar between patients having treatment for AS or AR. Patients with AR had a significantly higher left ventricular ejection fraction compared with patients with AS (56.00% [55.00, 59.25] vs. 49.00% [40.00, 55.00], *p* = 0.002); four AR patients had a left ventricular assist device implanted. The dimensions of the aortic annulus and sinus of Valsalva were significantly larger on pre-TAVI computed tomography (CT) scans in patients with AR compared to AS; however, ascending aorta diameters were similar.

**Table 1:** Patient baseline characteristics

	Total (N = 88)	AS (N = 20)	AR (N = 68)	*p* Value
Age, y	80.00 [72.83, 84.00]	81.50 [76.67, 83.78]	80.00 [71.00, 84.00]	0.309
Female	39 (44.3%)	13 (65.0%)	26 (38.2%)	0.063
NYHA functional class				0.063
I	3 (3.4%)	0 (0.0%)	3 (4.4%)	
II	22 (25.0%)	8 (40.0%)	14 (20.6%)	
III	55 (62.5%)	11 (55.0%)	44 (64.7%)	
IV	8 (9.1%)	1 (5.0%)	7 (10.3%)	
Euroscore II	3.79 [2.11, 7.13]	2.50 [1.76, 4.90]	4.39 [2.27, 7.51]	0.057
Society of Thoracic Surgeons (STS) score (NA = 8)	2.63 [1.71, 4.24]	3.10 [1.45, 3.92]	2.50 [1.78, 4.25]	0.973
Body mass index, Kg/m^2^	26.28 [22.70, 29.02]	27.03 [24.55, 29.23]	24.98 [22.68, 28.95]	0.312
Atrial fibrillation	39 (44.3%)	8 (40.0%)	31 (45.6%)	0.852
Chronic obstructive pulmonary disease	10 (11.4%)	0 (0.0%)	10 (14.7%)	0.155
Dyslipidemia	39 (44.3%)	8 (40.0%)	31 (45.6%)	0.852
Diabetes mellitus	17 (19.3%)	2 (10.0%)	15 (22.1%)	0.38
Hypertension	79 (89.8%)	18 (90.0%)	61 (89.7%)	1
Extracardiac arteriopathy	9 (10.2%)	1 (5.0%)	8 (11.8%)	0.647
Previous cardiac surgery	14 (15.9%)	3 (15.0%)	11 (16.2%)	1
Coronary artery disease	41 (46.6%)	10 (50.0%)	31 (45.6%)	0.926
Previous stroke	11 (12.5%)	2 (10.0%)	9 (13.2%)	1
Left ventricular ejection fraction, % (NA = 14)	50.00 [41.75, 55.00]	56.00 [55.00, 59.25]	49.00 [40.00, 55.00]	0.002
Left ventricular end-diastolic diameter, mm (NA = 18)	55.00 [15.35, 61.75]	48.00 [43.00, 52.00]	56.00 [7.10, 62.00]	0.299
Left ventricular end-systolic diameter, mm (NA = 19)	37.00 [5.90, 47.00]	26.00 [25.00, 33.00]	37.50 [5.62, 47.25]	0.373
Aortic valve area, cm^2^ (NA = 25)	1.80 [0.90, 2.40]	0.70 [0.60, 0.84]	2.20 [1.75, 2.70]	<0.001
Mean aortic valve gradient, mmHg (NA = 14)	9.50 [5.03, 23.25]	42.00 [36.50, 50.50]	7.00 [5.00, 10.00]	<0.001
Mitral regurgitation (NA = 1)				0.271
None or trace	33 (37.9%)	11 (55.0%)	22 (32.8%)	
Mild	11 (12.6%)	2 (10.0%)	9 (13.4%)	
Mild to moderate	18 (20.7%)	5 (25.0%)	13 (19.4%)	
Moderate	4 (4.6%)	0 (0.0%)	4 (6.0%)	
Moderate to severe	4 (4.6%)	1 (5.0%)	3 (4.5%)	
Severe	17 (19.5%)	1 (5.0%)	16 (23.9%)	
Previous pacemaker	10 (11.4%)	1 (5.0%)	9 (13.2%)	0.536
Left ventricular assist device	4 (5.1%)	0 (0.0%)	4 (6.9%)	0.537
Creatinine	0.97 [0.81, 1.60]	0.89 [0.78, 1.32]	1.08 [0.83, 1.81]	0.157
Glomerular filtration rate, ml/min/1.73 m^2^	57.69 ± 24.93	60.07 ± 23.15	56.99 ± 25.55	0.630
Chronic renal replacement	6 (6.8%)	2 (10.0%)	4 (5.9%)	0.891
Mean annular perimeter, mm (NA = 2)	81.05 [74.03, 85.45]	73.50 [71.45, 81.00]	82.55 [76.15, 86.22]	0.002
Perimeter-derived diameter, mm (NA = 2)	25.88 [23.65, 27.25]	23.40 [22.74, 25.78]	26.39 [24.30, 27.53]	0.001
Mean aortic annular area, mm^2^(NA = 2)	503.45 [420.25, 565.60]	416.30 [376.40, 502.98]	526.00 [442.70, 577.35]	0.001
Aortic annulus–Lca distance, mm (NA = 2)	14.47 ± 3.75	13.81 ± 2.68	14.67 ± 4.01	0.368
Aortic annulus–Rca distance, mm (NA = 2)	18.03 ± 3.89	19.66 ± 2.46	17.81 ± 4.01	0.242
Sinus of Valsalva diameter, mm (NA = 2)	36.94 ± 5.29	32.40 ± 2.77	37.29 ± 5.29	0.046
Sinotubular junction diameter, mm (NA = 16)	32.20 [29.98, 37.32]	30.60 [29.00, 31.10]	32.90 [30.10, 37.50]	0.08
Ascending aorta diameter (NA = 16)	37.65 [34.88, 40.45]	35.30 [34.90, 35.60]	37.90 [34.85, 40.80]	0.293

*Notes.* Values are n (%) or median [IQR].

Abbreviations: AR, aortic regurgitation; AS, aortic stenosis; IQR, interquartile range; NA, not available; NYHA, New York Heart Association class.

### Procedural Characteristics and In-Hospital Outcomes

Procedure details are shown in Table 2. All valves were implanted through transfemoral access (100%), and conscious sedation was used in 81 (92.0%) patients. Balloon predilatation was performed on 16 patients, all of whom had AS. Balloon postdilatation was performed in 5 (5.6%) patients, with one patient being treated for AR.

**Table 2:** Procedural characteristics

	Total (N = 88)	AS (N = 20)	AR (N = 68)	*p* Value
Access route (NA = 1)				
Transfemoral	87 (100.0%)	20 (100.0%)	67 (100.0%)	1
Valve size (NA = 1)				0.002
23 mm	15 (17.0%)	8 (40.0%)	7 (10.4%)	
25 mm	24 (27.3%)	7 (35.0%)	17 (25.4%)	
27 mm	48 (54.5%)	5 (25.0%)	43 (64.2%)	
Anesthesia (NA = 2)				0.856
General anesthesia	4 (4.7%)	1 (5.0%)	3 (4.5%)	
Conscious sedation	81 (94.2%)	19 (95.0%)	62 (93.9%)	
Predilatation (NA = 1)	16 (18.4%)	16 (80.0%)	0 (0.0%)	<0.001
Postdilatation (NA = 2)	5 (5.8%)	4 (20.0%)	1 (1.5%)	0.011
Procedure duration, min (NA = 2)	70.50 [59.00, 86.75]	69.00 [58.25, 80.00]	73.50 [59.00, 87.75]	0.477
Contrast, ml (NA = 1)	152.00 [114.00, 195.00]	156.00 [113.75, 181.75]	150.00 [115.50, 195.00]	0.675
Fluoroscopy time, min (NA = 1)	16.00 [12.65, 20.00]	16.00 [13.00, 20.58]	15.80 [12.05, 19.95]	0.565

*Notes.* Values are n (%) or median [IQR].

Abbreviations: AR, aortic regurgitation; AS, aortic stenosis; IQR, interquartile range; NA, not available.

In-hospital outcomes are reported in Table 3. There was no conversion to open heart surgery, no valve embolization, no major vascular complications, no in-hospital deaths, 3 (3.4%) new strokes, and 11 (12.6%) new pacemaker implantations. The median intensive care unit stay was 1.00 [1.00, 1.00] day. The mean aortic pressure gradient on predischarge echocardiography was significantly higher in patients with AS than in AR (AS: 6.00 mmHg [4.75, 8.00] and AR: 4.00 mmHg [3.00, 5.00], *p* = 0.001). The effective orifice area was similar between the two groups.

**Table 3:** Outcome characteristics

	Total (N = 88)	AS (N = 20)	AR (N = 68)	*p* Value
Conversion to open surgery	0 (0.0%)	0 (0.0%)	0 (0.0%)	1
Valve embolization	0 (0.0%)	0 (0.0%)	0 (0.0%)	1
In-hospital mortality	0 (0.0%)	0 (0.0%)	0 (0.0%)	1
Vascular complication				0.264
None	79 (90.8%)	19 (95.0%)	60 (89.6%)	
Minor vascular complication	6 (6.9%)	0 (0.0%)	6 (9.0%)	
Major vascular complication	2 (2.3%)	1 (5.0%)	1 (1.5%)	
New stroke	3 (3.4%)	1 (5.0%)	2 (3.0%)	1
Permanent pacemaker implantation	11 (12.6%)	0 (0.0%)	11 (16.4%)	0.12
Total ICU stay, d (NA = 3)	1.00 [1.00, 1.00]	1.00 [1.00, 2.00]	1.00 [1.00, 1.00]	0.061
Mean aortic valve gradient at discharge, mmHg (NA = 4)	5.00 [3.00, 5.25]	6.00 [4.75, 8.00]	4.00 [3.00, 5.00]	0.001
Aortic valve area at discharge, cm^2^ (NA = 21)	2.40 [2.05, 2.65]	2.35 [2.15, 2.60]	2.40 [2.05, 2.75]	0.712
Paravalvular regurgitation (Echo) (NA = 3)				0.197
None	54 (63.5%)	14 (70.0%)	40 (61.5%)	
Trace	24 (28.2%)	3 (15.0%)	21 (32.3%)	
Mild	7 (8.2%)	3 (15.0%)	4 (6.2%)	
Mild to moderate	0 (0.0%)	0 (0.0%)	0 (0.0%)	
Moderate	0 (0.0%)	0 (0.0%)	0 (0.0%)	
Moderate to severe	0 (0.0%)	0 (0.0%)	0 (0.0%)	
Severe	0 (0.0%)	0 (0.0%)	0 (0.0%)	
Regurgitant fraction (video-densitometry), % (NA = 41)	3.0% [1.0%, 7.0%]	5.0% [3.0%, 6.0%]	2.0% [0.0%, 7.0%]	0.21

*Notes.* Values are n (%), mean ± SD, or median [IQR].

Abbreviations: AR, aortic regurgitation; AS, aortic stenosis; ICU, intensive care unit; IQR, interquartile range; NA, not available.

### Core Lab Aortography Analysis

**Figure 1:**
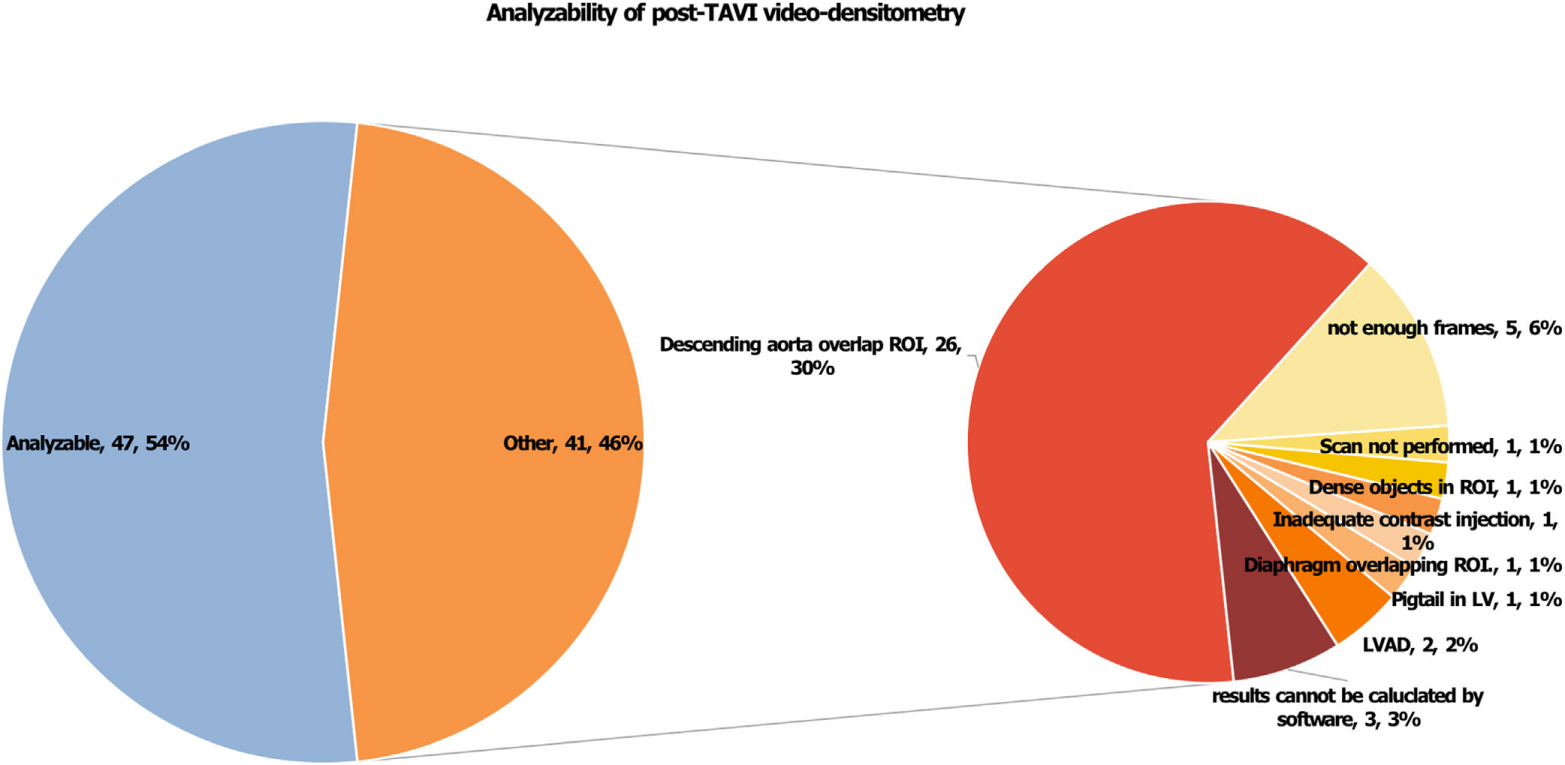
Analyzability of post-TAVI video-densitometry analysis. Abbreviations: LV, left ventricle; LVAD, left ventricular assist device; ROI, region of interest; TAVI, transcatheter aortic valve implantation.

Pre-THV aortograms were performed in 76 patients, with 17 (22.4%) suitable for analysis; the reasons for excluding the remaining 59 aortograms are shown in Supplemental Figure 1. Post-THV aortograms were performed in 87 patients, with 47 (54.0%) suitable for analysis; the main reasons preventing analysis were the descending aorta overlapping the outflow tract (30%), insufficient frame counts (6%), and unsupported file formats (4%) (Figure 1).

**Figure 2:**
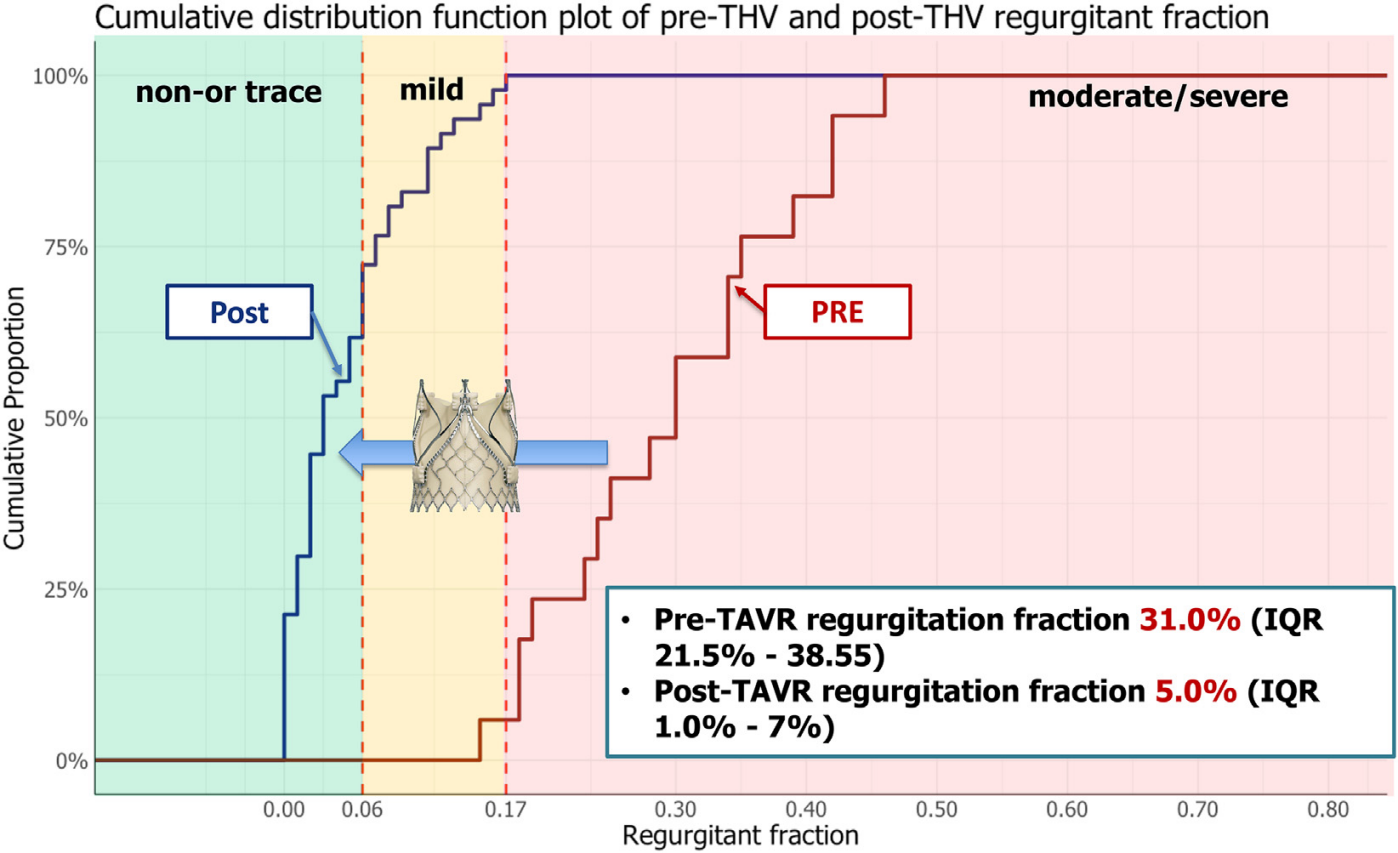
Cumulative distribution curves of pre- and post-THV regurgitant fraction on video-densitometry. Red curve indicates pre-THV. Blue curve indicates post-THV. Abbreviations: IQR, interquartile range; TAVR, transcatheter aortic valve replacement; THV, transcatheter heart valve.

The median regurgitant fraction pre-THV was 31% (interquartile range [IQR] 21.5-38.5%), while post-THV it was 5.0% (IQR 1.0- 7.0%), with no patients above the 17% threshold for more than moderate AR (Figure 2). In the 47 patients with post-TAVI angiography suitable for analysis, the incidence of none/trace AR was 72.3%, and mild AR was 27.7%, with no patients having moderate/severe AR.

**Figure 3:**
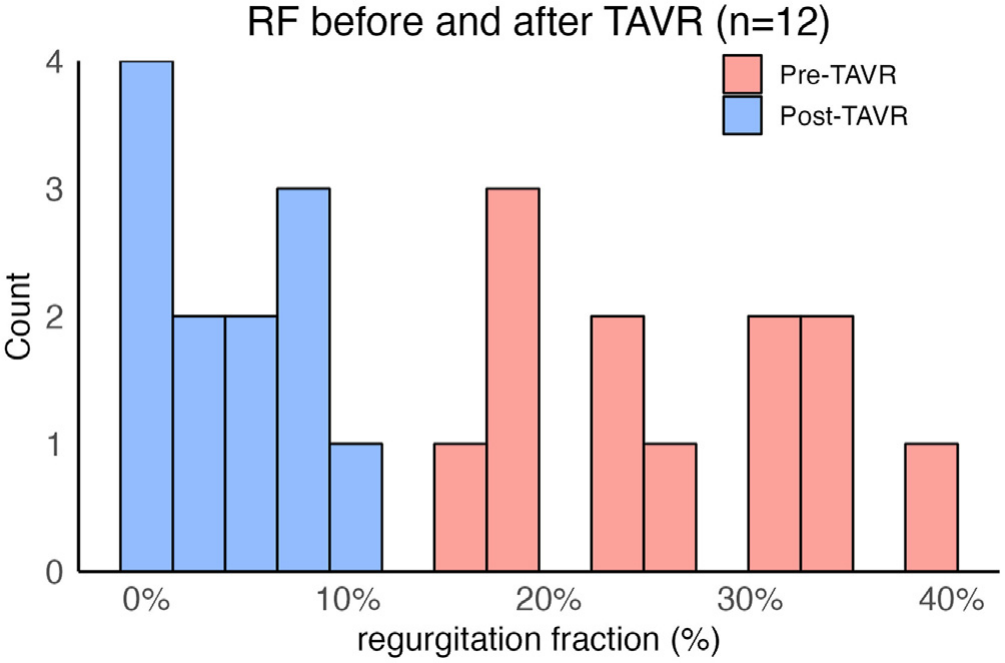
Paired analysis of pre- and post-THV video-densitometric RF. Abbreviations: RF, regurgitant fraction; TAVR, transcatheter aortic valve replacement; THV, transcatheter heart valve.

Analysis of the 12 cases with paired pre- and post-THV video-densitometry showed a significant reduction in the median RF from 24.5% (18.8-31.0%) to 3.5% (1.0-7.0%), *p* < 0.001, with an absolute reduction in RF of 21.8% ± 8.1% (Figure 3).

The video-densitometry analysis comparing patients with AS and AR is shown in Table 4. Pre-THV, the RF was only available in patients with AR, while the median post-THV RF was similar between patients with AR and AS (AR: 2.0% [IQR 0.0-7.0%] vs. AS: 5.0% [IQR 3.0-6.0%], *p* = 0.210), as were the incidences of none/trace and mild AR (*p* = 0.676).

**Table 4:** Core lab video-densitometry analysis

Regurgitant fraction (video-densitometry)	Total	AS	AR	*p* Value
Pre-TAVI (n = 17)	31.0% [21.5%, 38.5%]	NA	31.0% [21.5%, 38.5%]	NA
Post-TAVI (n = 47)	3.0% [1.0%, 7.0%]	5.0% [3.0%, 6.0%]	2.0% [0.0%, 7.0%]	0.21
Post-TAVI AR severity (video-densitometry)				0.676
None or trace	34 (72.3%)	9 (81.8%)	25 (68.4%)	
Mild	13 (27.7%)	2 (18.2%)	11 (30.6%)	
Moderate/severe	0 (0.0%)	0 (0.0%)	0 (0.0%)	

*Notes.* Values are n (%) or median [IQR].

Abbreviations: AR, aortic regurgitation; AS, aortic stenosis; NA, not available; TAVI, transcatheter aortic valve implantation.

Both video-densitometry and echo agreed that no patients had moderate/severe AR following THV treatment. The echocardiographic classifications of AR were similar between those with- and without video-densitometry suitable for analysis (*p* = 0.581) (Supplemental Table 1).

## Discussion

This is the first study to report the aortic RF pre- and post-TAVI in patients with severe AR or AS treated with the JenaValve Trilogy THV system using quantitative video-densitometry. The main findings of this study are:

1) The median post-THV RF was only 5.0% (IQR 1.0-7.0%).
2) The incidence of post-TAVI AR was exclusively none/trace or mild, with more patients with none/trace than mild AR.
3) There was no significant difference in the severity of post-TAVI AR between patients treated for severe AR or severe AS.

JenaValve’s Trilogy THV is the first dedicated device designed for treating AR and has also been approved for treating AS. Its unique clipping mechanism can secure the native leaflets, regardless of the presence of annular calcification, while its locators enable alignment with the aortic cusps, reducing incomplete apposition of the prosthesis to the aortic annulus, the primary mechanism of post-TAVI AR.^12^ In this study, the AR seen post-treatment with a JenaValve THV in a predominantly AR cohort was comparable to the best-in-class of the new generation THV in cases of AS. Importantly, these results were similar in patients with AS or AR, indicating that the JenaValve’s design can effectively mitigate post-TAVI AR regardless of the implant indication.^12^

The incidence of post-TAVI moderate/severe AR was markedly lower in our study when compared to previous experiences of off-label THV implantation for patients with pure AR. For example, in the Performance of Currently Available Transcatheter Aortic Valve Platforms in Inoperable Patients With Pure Aortic Regurgitation of a Native Valve (PANTHEON) study, the incidence of moderate/severe post-TAVI AR was 9.2 and 10.1% for self-expandable and balloon-expandable valves, respectively,^13^ while in the current study, there were no such patients. This performance is even superior to the new-generation THVs, which have demonstrated less moderate/severe post-TAVI AR compared to first-generation devices when treating patients off-label for pure AR.^5^^,^^14^^,^^15^ These encouraging results could be attributed to the external sealing skirts added to the latest iterations of valve designs. Schneeberger et al. demonstrated no moderate/severe AR in 9 patients treated with the self-expanding ACURATE neo2 device (Boston Scientific, MA), while Amat-Santos et al. reported a technical success rate of 94.7% and an 8.9% rate of residual moderate/severe AR using the Myval Octacor THV (Meril Life, Vapi, India).^16^^,^^17^ However, these studies derived their data from small cohorts in expert centers without using quantitative analysis.

Importantly, in this study, we utilized the unique advantage of video-densitometry and assessed, for the first time, paired pre- and post-THV aortograms in 12 cases where both were suitable for analysis. With these data, we quantified the marked reduction in RF (21.8% ± 8.1%) immediately after THV implantation. Thus, if performed prospectively with a standardized acquisition protocol, quantitative results can be obtained from procedural aortograms without the need for additional contrast injections.

The results of JenaValve’s trilogy THV in this mixed cohort are comparable with other studies where quantitative video-densitometry was performed in patients with severe AS using the latest generation THVs.^18^ Among the more than a dozen TAVI devices previously assessed using video-densitometry, the ACURATE neo2 (N = 120) and the Myval Octacor (N = 103) had the lowest incidences of moderate/severe AR at 1.7 and 1.9%, respectively. In the 20 patients with AS and the 68 with AR treated with JenaValve THV, there were no cases of moderate/severe AR. In addition, mild AR was seen in 27.7% of patients, compared to 20% with the Myval Octacor and 30.8% with ACURATE neo2.^18^^,^^19^ Recently, residual AR has emerged into the spotlight, with multiple reports demonstrating the association of even mild para-valvular leaks with poor prognosis.^20^^,^^21^ Thus, the incidence of mild AR needs to be carefully monitored and minimized as much as possible. The results of JenaValve’s trilogy THV suggest that it is a viable option not only for patients with AR but also for AS.

In patients with AS, balloon predilatation is required before implanting the JenaValve THV as the device lacks the mechanisms to stretch calcified stenotic leaflets; furthermore, when TAVI expansion is insufficient, postdilatations are performed and needed, irrespective of implant indication. In our cohort, postdilatation was more frequent in patients with AS than AR (20.0 vs. 1.5%, *p* = 0.011), but the frequency of postdilatation was numerically lower than the reports of the ACURATE neo 2 THV,^22^ which also requires predilatation before implantation. Furthermore, the procedure and fluoroscopy time and, importantly, stroke rates were similar in AS and AR patients.

The favorable acute performance of the JenaValve in terms of the severity of AR and in-hospital outcomes is very reassuring, considering this cohort includes elderly patients with multiple comorbidities, with a median age of 80 years and a median Society of Thoracic Surgeons score of 3.79%. These findings, as well as the long-term outcomes and especially the association of clinical outcomes with residual AR, need to be elucidated with future studies. The clinical safety and efficacy of the JenaValve THV system have been evaluated in the transcatheter aortic valve implantation in patients with high-risk symptomatic native aortic regurgitation [NCT02732704] (107 patients) trial and are being evaluated in the ALIGN-AS [NCT02732691] (92 patients) trial. Promising initial results of the transcatheter aortic valve implantation in patients with high-risk symptomatic native aortic regurgitation study were reported, with the incidence of more than mild AR being 0.6% at 30-day follow-up.^23^ However, neither of these studies quantitatively assesses post-TAVI AR; thus, further studies will still be required following these two pivotal trials to demonstrate the quantitative efficacy of the JenaValve THV.

### Limitations

The current study has several limitations. First, this is a retrospective cohort study without a standardized imaging acquisition protocol. Thus, the number of patients with analyzable video-densitometry was low. However, the low analyzability can be easily overcome with pre-TAVI CT guidance or angiography guidance following the “Teng’s rule”.^24^ In a previous study conducted in Yamaguchi, Japan, the application of CT guidance can increase the analyzability from 57.4% to 100%.^25^ In the multinational ASSESS-REGURGE [NCT03644784] registry, the analyzability was consistent regardless of region and guiding method (CT or Teng’s rule).^26^ Thus, the low analyzability seen in this retrospective cohort can be eliminated in prospective cohorts with a standardized acquisition protocol, producing analyzability between 92% and 100%.^27^ However, our study is still the first quantitative aortography analysis of consecutive patients for this novel device. In addition, the severity of AR, as evaluated with echocardiography, was similar to those where video-densitometry could or could not be analyzed. Second, the study was comprised of selected patients from one geographic area and was performed in expert centers. Thus, our results may not be fully generalizable to all practices. Importantly, none of our patients exhibit moderate or severe AR after THV implantation. Thus, the potential to investigate the ability of video-densitometry to differentiate mild vs. mild to moderate residual AR is limited. Third, we could only report in-hospital clinical outcomes as our focus was exclusively on testing the residual AR after JenaValve THV treatment.

## Conclusion

Quantitative arotography with video-densitometry confirms the low rates of AR and the large reduction in RF following the implant of Jenavalve’s Triology THV, irrespective of implant indication; however, these limited data need corroboration in prospective studies using standardized acquisition protocols.

## Ethics Statement

The study was conducted in accordance with the principles of the Declaration of Helsinki and good clinical practice. The study was approved by the ethical committee in each participating center.

## Funding

The quantitative aortography analysis of this project is supported by the restricted fund from JenaValve Inc., Irvine, CA.

## Disclosure Statement

H. R. Figulla is the cofounder, shareholder, and consultant of JenaValve Inc, Irvine, CA. S. Garg receives consultation with Biosensors. R. von Bardeleben has been a consultant and the recipient of lecture honoraria from Abbott Structural Heart, Boehringer Ingelheim, Cardiac Dimensions, Edwards Lifesciences, GE Health Systems, and Philips Healthcare. H. Wienemann reports travel grants from JenaValve. M. Adam reports personal fees from JenaValve, Abbott, Boston Scientific, and Edwards Lifesciences. P. W. Serruys reports consulting fees from SMT, Novartis, Xeltis, Merillife, and Philips outside the submitted work. The other authors had no conflicts to declare.
