# Supplementary material for: Quantitative Aortography Analysis of JenaValve’s Trilogy Transcatheter Aortic Valve Implantation System in Patients With Aortic Regurgitation or Stenosis
Source: Struct Heart. 2024 Jul 23;8(5):100346. doi: 10.1016/j.shj.2024.100346 (PMC11403081; doi:10.1016/j.shj.2024.100346)

| **SUPPLEMENTAL TABLE 1 Echocardiographic aortic regurgitation in patients with and without analysable aortography** | | | | | | |
| --- | --- | --- | --- | --- | --- | --- |
| **Echocardiographic regurgitation grade** | **None** | **Trace** | **Mild** | **Moderate/Severe** | **NA** | **P=0.581** |
| VD not analyzable | 26 (63.4%) | 10 (24.4%) | 2 (4.9%) | 0(0%) | 3 (7.3%) |  |
| VD analyzable | 28 (59.6%) | 14 (29.8%) | 5 (10.6%) | 0(0%) | 0(0%) |  |
| NA=not available | | | | | | |

**Supplemental Figure legend:**

**Supplemental Figure 1.** Analyzability of pre-THV video-densitometry analysis

**Supplemental Figure 1.**


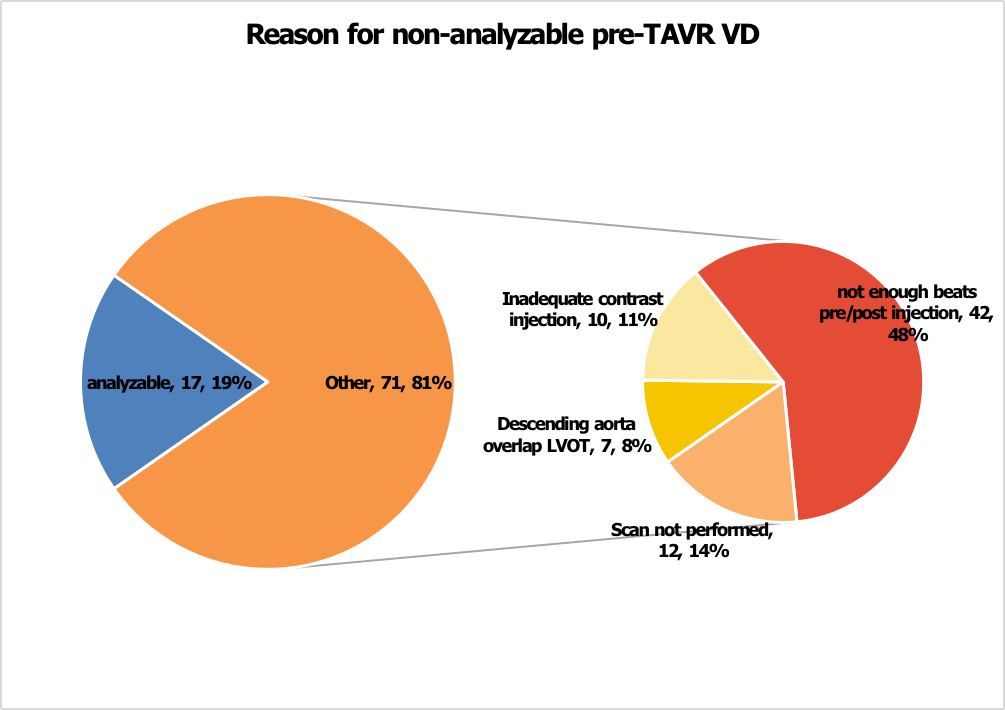

Supplement: Supplementary Table 1 and Figure 1 [file mmc1.docx]
